# Nuclear spin hyperpolarization of pyruvate enables longitudinal monitoring of treatment response in intestinal tumor organoids

**DOI:** 10.1002/mrm.70008

**Published:** 2025-07-30

**Authors:** Josh P. Peters, Hang Xiang, Charbel D. Assaf, Farhad Haj Mohamad, Philip Rosenstiel, Stefan Schreiber, Jan‐Bernd Hövener, Konrad Aden, Andrey N. Pravdivtsev

**Affiliations:** ^1^ Section Biomedical Imaging, Molecular Imaging North Competence Center, Department of Radiology and Neuroradiology, University Hospital Schleswig‐Holstein Kiel University Kiel Germany; ^2^ Institute of Clinical Molecular Biology Kiel University Kiel Germany; ^3^ Department of Internal Medicine I University Hospital Schleswig‐Holstein Kiel Germany

**Keywords:** hyperpolarization, intestinal carcinogenesis, organoids, pyruvate, rapamycin

## Abstract

**Purpose:**

Colorectal cancer, a leading cause of death in the Western world, is increasingly affecting younger populations. The Warburg effect, characterized by enhanced lactate production, is a hallmark of this cancer type. Although ^18^F‐FDG PET‐CT is commonly used for diagnosis, MRI offers higher spatial and chemical resolution without the drawbacks of radiation. However, MRI's low sensitivity has been a barrier to real‐time metabolic imaging, and hence its implementation in clinical practice. Hyperpolarization has significantly boosted NMR sensitivity, enabling detailed metabolic studies in vivo.

**Methods:**

This study uses hyperpolarized [1‐^13^C]pyruvate with dissolution dynamic nuclear polarization to noninvasively monitor metabolic changes in intestinal organoids from a genetically defined mouse model of spontaneous carcinogenesis (Rnaseh2b/Xbp1^ΔIEC^) with a previously established targeted therapeutic intervention (mTOR inhibition by rapamycin).

**Results:**

Hyperpolarized NMR revealed a 6.6‐fold reduction (*p* < 0.05) in lactate production in rapamycin‐treated organoids, indicating suppressed metabolic activity. This method also detected alanine and bicarbonate metabolism, highlighting its sensitivity. Unlike traditional methods that destroy cellular integrity, hyperpolarization enabled repetitive, noninvasive metabolic assessments.

**Conclusion:**

Hyperpolarized [1‐13C]pyruvate combined with NMR enables noninvasive, longitudinal monitoring of tumor metabolism in intestinal organoids while preserving cell viability and recultivation potential, bridging preclinical and clinical applications and affirming the method's potential for targeted metabolic imaging as a novel diagnostic and treatment control approach in cancer medicine.

## 1 INTRODUCTION

Colorectal cancer is one of the leading causes of death in the Western world and is shifting toward an earlier disease onset in life. Metabolic rewiring of the cancer epithelium toward increased lactate production from glucose and pyruvate, commonly described as the Warburg effect, is observed.^1^ The detection of increased glycolysis visualized with ^18^F‐FDG PET‐CT is widely established in diagnosing solid tumors. Clinical applications would greatly benefit from similar approaches, such as NMR spectroscopy and MRI, due to their increased spatial tissue resolution, chemical resolution, and non‐radiating imaging, allowing repetitive imaging during diagnosis and treatment periods.

NMR and MRI are gold standards for noninvasive metabolic profiling.^2^, ^3^ However, imaging in real time and with submicromolar sensitivity is challenging due to the low sensitivity of MRI. The introduction of nuclear spin hyperpolarization boosted the sensitivity of NMR by orders of magnitude, enabling real‐time molecular imaging even in vivo,^4^, ^5^, ^6^ such as for diagnosing pancreatic adenocarcinoma.^7^

Cells are useful phantoms for metabolic analysis. The transition from two‐dimensional to three‐dimensional (3D) cell structures better approximates the in vivo biochemistry.^8^, ^9^, ^10^ Organoids are clusters of organ‐specific cells in a 3D structure cultured in a microplate that can undergo self‐renewal.^11^, ^12^ Compared with traditional cellular models, an organoid contains multiple differentiated cell types derived from a stem cell. These features make organoids an ideal system for disease modeling and mechanism exploration. However, current methods of cancer metabolism tracking are limited to ^13^C‐tracing methods coupled to LC–MS, which requires the breakdown of cellular integrity and, therefore, the interruption of the biological process.

Transcription‐associated mutagenesis has recently been attributed to the development of somatic cancers in eukaryotes.^13^ It describes a compensatory TOP1‐mediated DNA repair mechanism at sites of increased frequency of genome‐embedded ribonucleotides, resulting in increased insertions‐deletions. Mechanistically, genome‐embedded ribonucleotides result from impaired ribonucleotide excision repair, which removes faulty DNA‐incorporated ribonucleotides during every round of cellular replication via the enzyme RNase H2.^13^ We have previously established mouse models of epithelial‐specific deletion of *Rnaseh2b* in murine intestinal epithelial cells (*Rnaseh2b* ^ΔIEC^) and subsequent double deletions (*Rnaseh2b/p53* ^ΔIEC^, *Rnaseh2b/Xbp1* ^ΔIEC^) to show the impact of ribonucleotide‐excision repair in the prevention of intestinal carcinogenesis.^14^, ^15^ Mechanistically, we delineated within the *Rnaseh2b/Xbp1* ^ΔIEC^ mouse model that malignant stem cell proliferation depends on the mTOR pathway, which can be successfully intercepted by mTOR inhibition in both in vivo and intestinal organoids.

**FIGURE 1:**
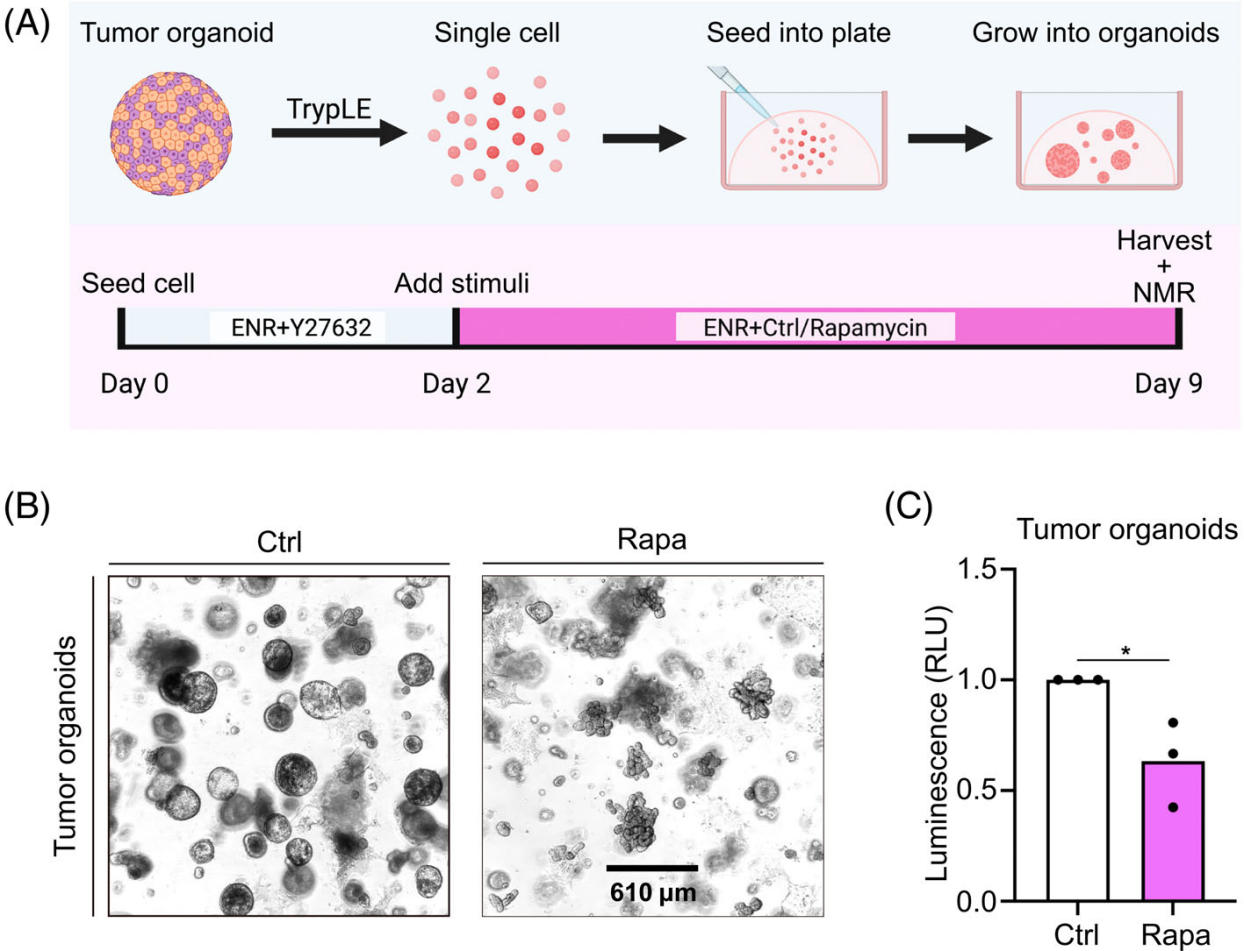
Rapamycin suppresses the proliferation of tumor organoids. (A) Sample preparation workflow: Dissociated tumor organoids were cultured with ENR + Y27632 for 2 days, then treated with DMSO (control [Ctrl]) or 1 μM rapamycin (Rapa) for 7 days. Subsequent analysis included NMR spectroscopy or CellTiter‐Glo assays. (B,C) Representative images (scale bar = 610 μM) (B) and CellTiter‐Glo assay (C) of Ctrl and Rapa tumor organoids groups of three individual replicates. The control group was normalized as 1, and significance was determined with an unpaired Student's t‐test (*p = 0.0305).

In this study, we used small intestine organoids isolated from healthy *Rnaseh2b/Xbp1* ^fl/fl^ mice (wild‐type [WT] hereafter) and spontaneous tumors from *Rnaseh2b/Xbp1* ^ΔIEC^ mice as the models to explore the changes in the metabolism of pyruvate as a result of their treatment with rapamycin to restrain cellular proliferation via the mTOR inhibition (Figure 1). Subsequently, we showed that hyperpolarized by dissolution dynamic nuclear polarization (dDNP) [1‐^13^C]pyruvate can be coupled with NMR to functionally discriminate different metabolic states between treated versus untreated tumor organoids. Hyperpolarization revealed a fewfold reduction of lactate production in response to rapamycin treatment and enabled the longitudinal study of organoids.

## 2 METHODS

### 2.1 Organoid culture

WT organoids were isolated from *Rnaseh2b*/*Xbp1* ^fl/fl^ mouse ileum. The tumor organoids were isolated from a spontaneous tumor *Rnaseh2b*/*Xbp1* ^ΔIEC^ mouse as described previously.^15^ Both WT and tumor organoids were cultured with Matrigel (Corning) with conditional hEGF (human epidermal growth factor), Noggin, and R‐spondin (often R‐spondin 1) medium (ENR medium).

### 2.2 Sample preparation

#### 2.2.1 Tumor organoid

The organoids were dissociated into single cells using TrypLE Express (Gibco, Thermo Fisher Scientific) and counted using a cell counter (LUNA‐II). A total of 10 000 cells per well were seeded onto a 24‐well plate with Matrigel. The seeded cells were cultured with conditional ENR medium with 10 μM/L Y27632 (STEMCELL Technologies) for the first 2 days. Then, they were cultured in a standard ENR medium containing DMSO or 1 μM rapamycin (LC Laboratories). Previously, we demonstrated that 1 μM rapamycin effectively suppressed the mTOR pathway and reduced hyperproliferation in H2b/Xbp1^ΔIEC^ organoids.^15^ Hence, the same concentration was selected here. After 7 days of treatment, the organoids were examined using the cell viability assay or NMR measurement.

#### 2.2.2 WT organoid

Organoids were passaged according to the same ratio and seeded from the same amount of well to guarantee the consistency of each experiment. During passaging, WT organoids were pipetted 100 times to break them into small pieces and seeded onto plates with Matrigel. Density was measured by microscopy to ensure it matched that of tumor organoids. After 5 days of culturing, organoids were continued by NMR measurement.

### 2.3 Organoid viability assay

Organoid viability was processed with CellTiter‐Glo 3D reagent (Promega Corp.) according to the manufacturer's protocol, and the luminescence was measured by a microplate reader (Tecan Infinite M Nano).

### 2.4 Staining of Paneth cell and stem cell

#### 2.4.1 Paneth cell staining

Organoids were passaged by pipetting and seeded on an eight‐well chamber. Rapamycin treatment was administered on the next day and continued for 4 days. Organoids were fixed with 4% PFA (paraformaldehyde) for 20 min at room temperature then washed by immunofluorescence (IF) buffer (phosphate‐buffered saline [PBS] + 0.2% Triton +0.05% Tween). After permeabilizing by permeabilization buffer (PBS + 0.5% Triton X‐100), organoids were blocked with 2% bovine serum albumin in IF buffer for 30 min. The primary antibody (rabbit anti‐lysozyme C, DAKO, EC 3.2.1.17, 1:500 dilution) was continuously incubated overnight at 4°C. After incubating the secondary antibody (Donkey anti‐Rabbit, Invitrogen A3290, 1:1000 dilution) for 1 h and after a 10‐min incubation of DAPI (Southern Biotech, SBA‐0100‐20, 1:1000 dilution), the whole slide was mounted with a fluorescent mounting medium (DAKO, S3023) followed by microscopy.

#### 2.4.2 Stem cell staining

Organoid stem cells were processed with the Click‐IT EdU assay (Click‐iT EdU Alexa Fluor 488 Imaging Kit, Invitrogen). On the day of staining, organoids were cultured with 10 μM EdU for 6 h before fixation. After fixation and permeabilization according to the IF protocol described previously, the stem cells were then stained with Click‐IT reaction cocktails. Nuclear staining and mounting proceeded as described previously.

### 2.5 Microscopy analysis

Organoids were visualized using the THUNDER imager 3D assay system, which is based on a Leica DMi8 microscope. A PLAN 5×/0.12 dry objective lens and a Leica MC190 HD Microscope Camera were used. The images were processed with the Leica *LAS X* software, and the confluency/area of the organoids was evaluated using the software's analysis feature.

### 2.6 Organoids preparation for NMR

All solutions and materials were cooled and coated with 1% bovine serum albumin to prevent the loss of organoids. The medium was removed from all wells, and 1.2 mL cold PBS was added to Wells 1, 3, and 5. The pipette tip disrupted the organoid domes in these cells until the mixture was homogenized and then transferred to Wells 2, 4, and 6, respectively. The final mix from six wells was collected and placed on ice for 3–5 min before centrifuging. The supernatant was carefully discarded, and 1 mL of cold PBS was added before centrifugation. Again, the supernatant was discarded, and 100 μL of cold PBS was added. An NMR tube was prepared with 150 mg of 0.5‐mm glass beads, followed by 60 μL PBS to fill the voids between the glass beads. Finally, the organoids were placed on the top of the glass beads.

### 2.7 Hyperpolarization

All dDNP experiments were performed using a cryogen‐free dDNP system (SpinAligner, Polarize) at about 1.4 K and 6.7 T as detailed previously.^16^ The samples contained 14 M [1‐^13^C]pyruvate (Sigma‐Aldrich, CAS: 99124‐30‐8) and 30 mM trityl radical (AH111501, POLARIZE). A microwave frequency between 187.02 and 187.105 GHz with 25 mW power was used for the polarization. For each dDNP experiment, 20 μL of the sample was taken from the stock, filled into the sample cup, and lowered into the microwave cavity. DNP was initiated, and the buildup in the solid state was monitored every 2 min with a ^13^C radiofrequency pulse of 1° for about 90 min; the observed characteristic polarization buildup time was about 20 min. After dissolution with 3.8 mL of superheated (˜200°C, 11 bar) dissolution medium containing 40 mM Tris‐buffer, 0.27 mM EDTA, and 50 mM NaCl to buffer the solution, remove any impurities, and adapt a physiological osmolality, the sample was transferred to an NMR system and detected 15 to 40 s later. The radical was not filtered. The injected solution consisted of 90–100 mM pyruvate and 0.16 mM trityl radical, diluted to 52–58 mM pyruvate and 0.09 mM trityl radical in the final mixture with the organoids.

### 2.8 NMR experiments

^13^C NMR signals were acquired using a 1 T ^13^C benchtop NMR (SpinSolve Carbon, Magritek) and a 9.4 T wide‐bore NMR with a narrow‐bore 5‐mm BBFO probe (WB400, Avance NEO, Bruker).

At 1 T, hyperpolarized spectra were acquired every 6 s with a 5° excitation angle (*α*) and a receiver gain of 31 dB. Thermally polarized, ^13^C‐enriched samples were averaged over 1800 to 3600 scans with *α* ˜ 90°, repetition time (3 s), and doped with the addition of 4 μL gadolinium (Gadovist, Bayer).

At 9.4 T, hyperpolarized spectra were acquired every 3 s with *α* ˜ 5°, ^1^H decoupling, and a receiver gain of 10. Thermally polarized samples were averaged over four scans (labeled substrates) with *α* ˜ 90° and 101 receiver gain.

### 2.9 Organoid reseeding

After the DNP experiment and subsequent NMR measurements, we sometimes extracted the organoids from the DNP solution by pipetting them from the NMR tube into an Eppendorf tube and centrifuging it. The solution was extracted, and the organoids were resuspended in a PBS buffer. Following the extraction, the organoids were washed with cold PBS and reseeded to a 24‐well plate with Matrigel cultured with conditional ENR medium containing DMSO or 1 μM rapamycin according to the original condition.

### 2.10 Analysis of NMR data

An exponential apodization of 1 Hz was applied for the kinetic spectra and 3 Hz for polarization analysis. A third‐order polynomial baseline correction was applied for each metabolite peak. Phase correction was applied in all cases. Integrals were used to quantify the signals (MNova, 14.2.2, Mestrelab Research S.L.). The integrals of each substrate and up to four products were saved in a .csv file for further kinetics analysis. The metabolism kinetics were analyzed using the protocols described in Peters et al.^17^

### 2.11 Analysis guide for organoids

A *Python* program was written to fit the first‐order kinetics of all metabolites regarding the substrate. This was done using the Broyden–Fletcher–Goldfarb–Shanno algorithm to minimize the system of ordinary‐differential‐equations for the substrate and up to four products. This leads to more accurate estimates of relaxation time, T_1,real_. As we do not extract the cell uptake rates, the fitted exchange rates are a superposition of uptake and metabolism. If specific metabolites were not observed, k was set to 0 inside the program. In some cases, the fit was improved by altering the penalty for residuals in the fit for the metabolites. The fitted variables were saved, and the fitted data were plotted for visual quality control. The program is detailed in Peters et al.^17^

### 2.12 Quantification of polarization

NMR spectra after dissolution were measured in parallel on a 1T benchtop NMR in a standard NMR tube without organoids and on a 9.4 T NMR in a tube with organoids containing glass beads at the bottom, to keep the organoids in the sensitive area of the coil.

**FIGURE 2:**
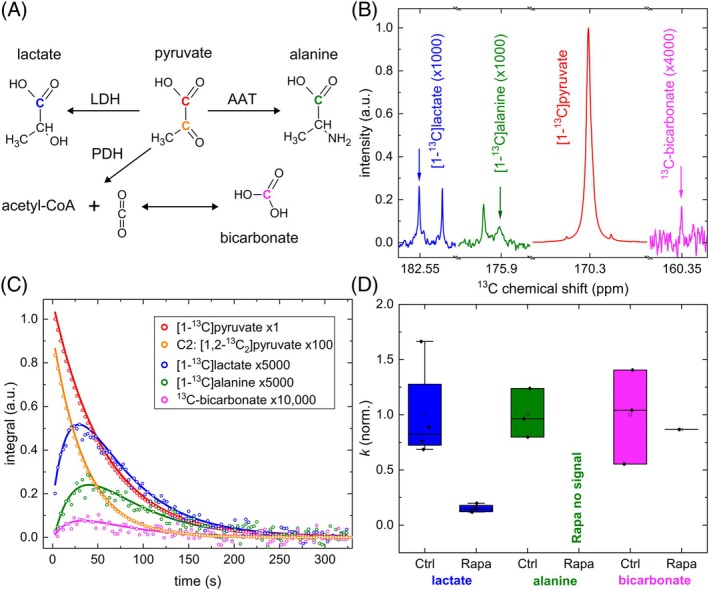
Hyperpolarization‐enhanced NMR revealed the strong metabolic effect of rapamycin treatment. (A) Chemical diagram of pyruvate metabolism: pyruvate with ^13^C labeled C1 (*red*) via enzymatic reactions becomes lactate (*blue*), alanine (*green*), CO_2_, and bicarbonate (*purple*). (B) The metabolic processes are revealed with hyperpolarization‐enhanced real‐time ^13^C NMR measurements. (C,D) The fitted kinetics of corresponding signals of pyruvate and three products (C) showed significant changes in conversion rate constants for the production of lactate and alanine (D). The low signal‐to‐noise ratio (SNR) of the bicarbonate signal did not allow us to make such a conclusion, and bicarbonate was observed only in one instance in the Rapa group. The *k*‐rate for lactate as an anaerobe metabolism marker is significantly lower in Rapa group (*p* = 0.016, 6.6 times, effect size *d* = 2.7). No alanine signal was observed in the Rapa group and only in three cases for the control (Ctrl). This suggests the same or more significant difference in signal between the Ctrl and Rapa groups when compared with lactate, as the alanine in the treated group is hidden within the noise floor (SNR of alanine in the control group is about 5). A substantial difference in signal and SNR can be observed between the different metabolites (B), where spectra of metabolites at their maximum intensities are shown. No CO_2_ is observed due to a sample pH of about 7.6^***19***^ (*N* = 4 for Ctrl and Rapa groups). The average ^13^C polarization at the beginning of measurements was (26.9±5.7)% for *n* = 8. A 1 T and 9.4 T NMR spectrometer were used for data acquisition. AAT, alanine aminotransferase; LDH, lactate dehydrogenase; PDH, pyruvate dehydrogenase.

**FIGURE 3:**
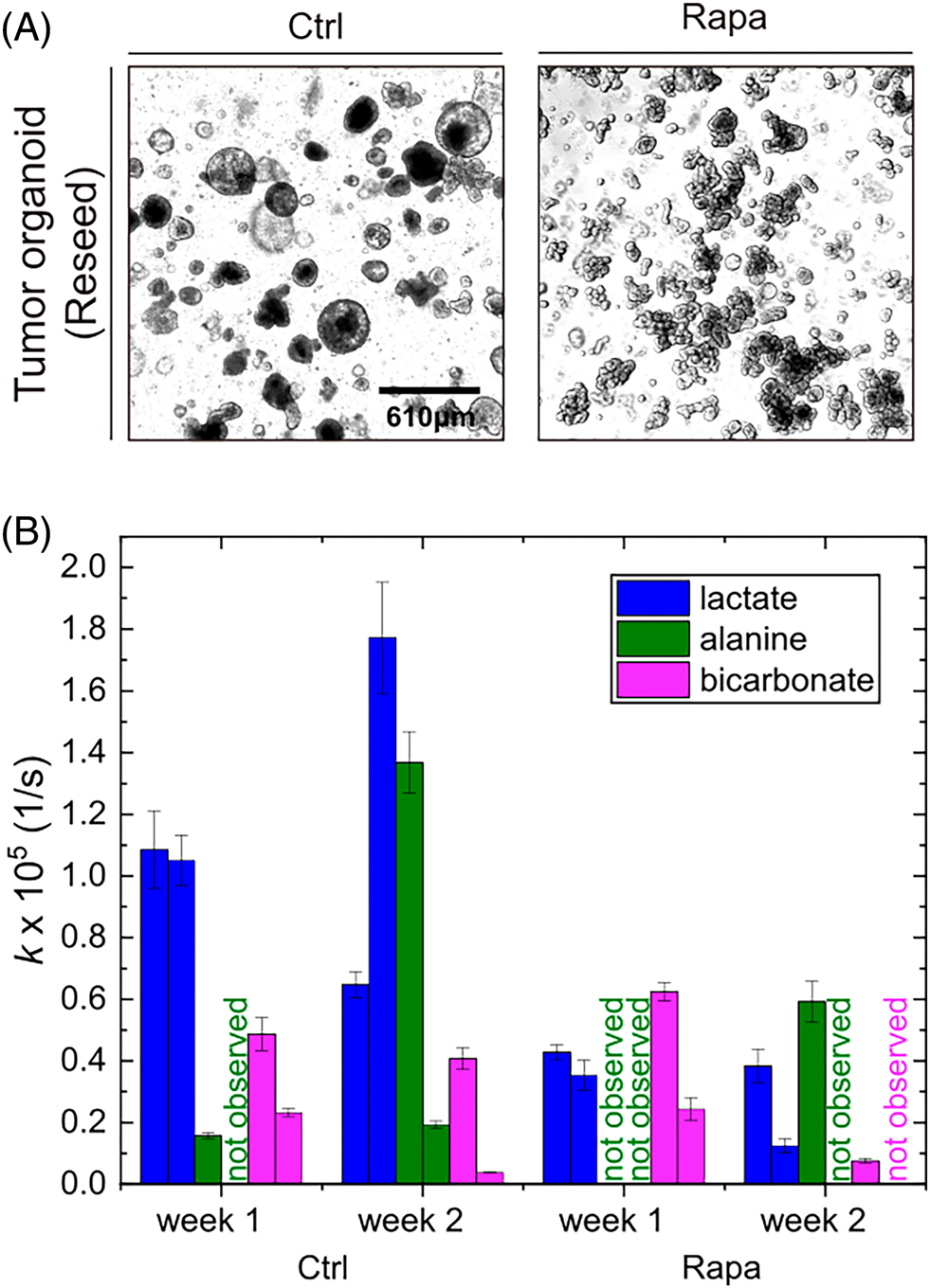
Hyperpolarized NMR and reseeding of organoids enabled longitudinal metabolic observation. Two pairs of control and rapamycin‐treated organoids were prepared and measured as before. After the first measurement, the organoids were reseeded and incubated for 1 more week, followed by a subsequent second NMR experiment. (A) Representative images were taken on day 3 of reseeding. Both control (Ctrl) and Rapa groups maintained their original morphological phenotypes. Scale bar = 610 μm. (B) In most cases, a decrease in lactate and bicarbonate and an increase in alanine metabolism can be observed from week 1 to week 2. The rise in alanine metabolism is expected to result from cell aging. Note that we split the second Ctrl sample after the first measurement because of the high cell density. Thus, it continued to grow more intensely than the other organoids, leading to an increased lactate metabolism in week 2, showcasing the importance of organoid handling during longitudinal experiments. The average ^13^C polarization at the beginning of measurements was (35.6 ± 2.5)% for *n* = 8. A 1 T and 9.4 T NMR spectrometer were used for data acquisition.

The signal of the first hyperpolarized spectrum at 1 T was used to calculate the liquid state polarization after sample transfer from dDNP to 1T NMR. As a reference, a thermally polarized spectrum from the same sample at 1 T was measured subsequently. Liquid state polarization was not measured at 9.4 T in the same way, because the organoids constantly consumed the substrate inside the NMR. Therefore, the reference thermally polarized spectrum measured after the experiment will not reflect the signal of pyruvate adequately, as it will be partially converted to other metabolites, including volatile CO_2_. Because no metabolic reaction took place in the case of the parallel 1 T benchtop, we consider it the gold standard. Polarization quantified at 1 T correlates with the polarization value of the first spectrum on the 9.4 T system, which was measured in parallel. To account for the differences in transfer time between the 1T and 9.4T machines, we used the T_1_ at the lower field, closer to the transfer conditions, to deduct the polarization at the time point of measurement for the organoids (Table S2). Considering the long T_1_ at 1 T (71.5 ± 10.7 s, *n* = 18) and difference of transfer time for two spectrometers (5.9 ± 9.2, *n* = 18), the correction factor is not very significant. Please note, the measurement series presented in Figures 2 and 3 had some repairs of the DNP system in between, which account for the differences in observed polarization.

### 2.13 Statistics and reproducibility

All statistical analyses were performed in *OriginPro* 2024. The data were analyzed by a one‐sided two‐sample t‐test, with the null hypothesis (H0): mean(Ctrl)−mean(Rapa)≤0. A value of *p*‐value less than 0.05 was considered statistically significant. Equal variance assumed and not assumed (Welch correction) were compared. The effect size was evaluated using Cohen's D using the difference of means and the pooled standard deviation of both groups. All measured estimate parameters for *k*, area under the curve (AUC), max%, *t*_max_ are given in Table S1 as well as the estimated polarization during the measurements in Table S2.

## 3 RESULTS

### 3.1 Cancer treatment effect

The successful inhibition of tumor growth after rapamycin treatment was confirmed using microscopy (Figure 1B) and CellTiter‐Glo assay to measure cellular ATP production (Figure 1C). In line with previous studies, we observed a significant reduction in cell growth and cellular ATP production in response to rapamycin treatment.

Following the completion of growth, we removed the organoids from their Matrigel on which they were grown and resuspended them in physiological PBS buffer inside the NMR tube containing glass beads.^17^, ^18^ The hyperpolarized juice, containing [1‐^13^C]pyruvate, was injected into the NMR tube, followed by the NMR measurement.

Using this protocol, we examined four pairs of control and rapamycin‐treated tumor organoids and observed rapid metabolism (Figure 2). The sensitivity was high enough to observe lactate, alanine, and bicarbonate. The pH and ^13^C pyruvate polarization was 7.4 ± 0.3 and (27.6 ± 6.3)% for the control and 7.4 ± 0.4 and (26.3 ± 4.9)% for the stimulated organoids, respectively. No CO_2_ signal was found due to a significant shift toward bicarbonate at neutral pH.^19^ The coverage area of organoids was (24.6 ± 3.5)% for the control and (20.8 ± 4.5)% for the treated organoids in each well, while six wells were combined for one experiment.

The conversion rate (*k*), AUC, and maximum signal intensity (max%) for the metabolic product lactate were significantly different (*p* < 0.05, not assuming equal variance) between both groups: The metabolism (*k*) was slower by 6.6 in treated organoids, which also led to an approximate 6.2‐times reduced lactate signal. The effect size, measured by Cohen's *d*, was substantial, with values between 2.7 and 3.9, indicating both statistical and practical significance in the observed differences. The time from the start of metabolism to maximum lactate signal intensity (*t*_max_) was 5.2 s faster in the control group. However, the difference was not significant (*p* = 0.1; Figure S1). A higher number of measurements would be needed to confirm this. Alanine metabolism was observed in the control group only.

Bicarbonate was observed in the treated organoids only once, while it was readily observed in the control group. The low signal‐to‐noise ratio of about 1.7 was not sufficient for a reliable analysis, indicated by an increased standard deviation of the results.

### 3.2 Tumor‐induced metabolic changes

To further strengthen our findings, we compared the metabolism of tumor organoids to WT intestinal organoids. As expected, we observed increased conversion rates of [1‐^13^C]pyruvate to lactate, alanine, and bicarbonate in tumor control organoids compared with WT organoids. However, following the treatment of tumor organoids with rapamycin, reduced metabolism below WT metabolism was observed (Figure S2A).

### 3.3 Demonstration of longitudinal metabolomics

To demonstrate the non‐destructiveness of NMR, we conducted an experiment in which intestinal tumor organoids were reseeded after the NMR measurement and incubated for another week, followed by an additional NMR measurement (Figure 3).

First, we prepared two pairs of control and rapamycin‐treated organoids and measured them as before (the same process as in Figure 1). Subsequently, after the first NMR measurement, the cells were extracted and recultivated in Matrigel and incubated for 1 more week with or without treatment. The organoids remain viable after both NMR measurements: We found a decrease in lactate and bicarbonate metabolism, whereas the alanine metabolism increased in both groups after an additional week of incubation (Figure 3). The production of lactate and alanine was higher in the control group than in the treated organoids in both age groups.

After reseeding, we split one control group due to a very high cell density (second control sample, Figure 3B), leading to accelerated growth and increased lactate metabolism in Week 2.

We found that not only tumors but also WT organoids, which are considered to be less resilient compared with tumorous ones, can be reseeded. Moreover, the reseeded WT organoids were able to be passaged.

## 4 DISCUSSION

### 4.1 Cancer treatment effect

A higher organoid's coverage area was expected for the control (Figure 1C), as the growth was impaired in the stimulated group. However, the difference in coverage alone does not explain the difference in metabolism, suggesting a substantial reduction of metabolism per cell.

A signal‐to‐noise ratio of about 5 for alanine in the control group indicates that max% of alanine is at least 5 times lower in the treated compared with the control group. Because max%, AUC, and *k* follow the same trends (Figure S1), this observation also suggests that *k* and AUC are at least 5 times lower in the treated group, indicating an equal to lactate dehydrogenase reduction of alanine aminotransferase activity in the group treated with rapamycin.

A comparison of the lactate‐to‐bicarbonate ratios between the treated and control groups would be feasible to quantify the ratio of anaerobic/aerobic metabolism. However, this was not possible, as bicarbonate was observed in the treated organoids only once due to high treatment efficacy.

### 4.2 Comparison of tumor and WT organoids

Increased conversion rates of [1‐^13^C]pyruvate to lactate, alanine, and bicarbonate in tumor control organoids compared with WT organoids before treatment and reduced metabolism following the treatment of tumor organoids with rapamycin indicate that the rapamycin treatment efficiently suppresses metabolism below the level of the WT organoids. These metabolic changes can potentially be used as a marker for the successful treatment.

Interestingly, despite morphological and metabolic proximity to WT, rapamycin‐treated tumor organoids maintained tumor characteristics: They exhibited a high density of stem cells, with no observable Paneth cells. However, both cell types occurred in the crypt‐like domain in WT organoids (Figures 2 and S3).

### 4.3 Persistence of rapamycin treatment

Cell line studies demonstrate that mTOR pathway inhibition reverses quickly (within 10 min) following rapamycin removal.^20^ In our case, organoids were measured (Figure 2) following removal from rapamycin‐enriched medium and Matrigel. During removal, the cells were at 4°C, followed by 14 min inside the NMR spectrometer warming up to 37°C. Small standard deviations for conversion rate constant to lactate indicate that, in our case, the effect from the removal was not very strong. However, the persistence of reduced lactate levels following rapamycin withdrawal was not tested in our study. Clinical trials consistently show that rapamycin's antitumor effects (like tumor regression) are not sustained after treatment cessation, leading to tumor regrowth, and necessitating continuous administration.^21^, ^22^ Given that lactate modulation is linked to mTOR activity, this rapid reversal of the target pathway and the clinical dependence on continuous treatment strongly suggest that the reduction in lactate levels achieved with rapamycin treatment is unlikely to persist significantly after withdrawal and is short‐lived.

### 4.4 Effect of long organoid cultivation

Overall, the observed metabolic changes are in line with the data presented previously.^14^, ^15^ However, in our case, we used noninvasive hyperpolarization‐enhanced NMR, providing the change for longitudinal studies,^23^ which was suggested but not proven previously for organoids.^9^

Increased alanine metabolism in both groups after an additional week of incubation may be due to stress or age‐related cell degradation. The increase in lactate metabolism in the split control group indicates that the protocol of organoid handling during reseeding may affect the organoids more than the stress from the experiment itself. This observation does not oppose repeatability in longitudinal studies but highlights the importance of standardized organoid handling.

The ability to reseed and passage even WT organoids indicates their capacity for long‐term culture (Figure S2B,C) and shows that the reseeding procedure is quite gentle on the cells. Future improvements to the protocol may further decrease the effect of reseeding on the cells, leading to increased reproducibility.

## 5 CONCLUSION

We showed that ^13^C NMR with hyperpolarized [1‐^13^C]pyruvate is a non‐destructive method enabling repetitive longitudinal monitoring of tumor metabolism in intestinal organoids. A few technical limitations should be considered concerning the handling of organoids. Ideally, one should not remove cells from Matrigel for the experiment to minimize stress.^23^, ^24^ However, for the scope of the hyperpolarized experiment, it is necessary to have a good perfusion of the DNP solution in the cells. In other studies, NMR bioreactors with immobilized cells and a peristaltic pump supplying the medium^25^, ^26^, ^27^ were designed to continuously circulate nutrients or cells for NMR measurements. However, such systems cause sheer stress that may reduce cell viability and lead to cell rapture or detachment.^28^ This is particularly relevant for shear‐sensitive cells^29^ and organoids.

Using our approach, we found that the organoids had good viability inside the NMR tube despite the washing procedure to eliminate the Matrigel. This is highlighted by three aspects: strong metabolism confirmed by ^13^C NMR, intact cells when checked with Trypan blue after the experiment, and successful reseeding and passaging after the first and second NMR experiments.

Overall, our data demonstrate that hyperpolarized [1‐^13^C]pyruvate can be coupled with NMR to monitor tumor metabolism in intestinal organoids longitudinally. This is particularly relevant, as recultivation is typically impossible with current state‐of‐the‐art methods to monitor cellular metabolism, which destroys cells to access metabolites. Luckily, hyperpolarization methods are rapidly developing, enabling new substrates to be applied for tracing metabolism and making hyperpolarization more accessible.^30^, ^31^, ^32^, ^33^, ^34^ In summary, we showed that hyperpolarization allows for noninvasive metabolic monitoring of cancer before and after treatment and at different ages of cells, putting a bridge between in vitro preclinical studies and clinical applications.

## CONFLICT OF INTEREST STATEMENT

The authors declare no conflict of interest.

## Supporting information


**Figure S1.** Strong metabolic effect of rapamycin treatment revealed by hyperpolarization‐enhanced NMR. Four data sets were evaluated in both cases (four control, four treated with rapamycin). (A) The *k*‐rate for lactate as anaerobe metabolism marker is significantly lower in the treated tumor organoids (6.6 times, effect size *d* = 2.7). No alanine signal was observed in the treated tumor organoids, suggesting that alanine experiences also about the same difference in signal or more between control and treated when compared with lactate, as the alanine in the treated group is hidden within the noise floor (signal‐to‐noise ratio [SNR] of alanine of about 5 in the control group). Bicarbonate was only found in one instance for the treated organoids. This suggests a higher average metabolism for the control group in all cases. (B) The areas under kinetic curve (AUC) (Figure 2) closely match the results (treated 5.3 times lower, *d* = 3.9) from (A), as do the maximum signal intensity (C) treated 6.2‐times lower, *d* = 3.5). The maximum bicarbonate signal was found to be just above.
**Figure S2.** Comparison of pyruvate to lactate conversion rate, *k*, in wild‐type (WT), tumor control, and tumor rapamycin‐treated organoids and their morphology. (A) Comparison of the metabolism of tumor organoids to WT organoids. The WT organoids were enriched to increase the density of cells in the Matrigel. Without enriching, we could not readily observe their metabolism using hyperpolarization. The densities inside the Matrigel were 18.4% (WT), 34.8% (control), and 21.7% (stimulated). When comparing the conversion rates of Rapa and WT groups, the k of the WT organoids was higher, despite its lower density. This indicates that the rapamycin treatment was efficiently suppressing metabolism below the level of the WT organoids, indicating the success of the treatment. Polarization across all experiments was (28.6 ± 6.0)%, and pH in the NMR tube was 7.4 ± 0.4. (B) The WT organoids were reseeded after testing, and the representative pictures were taken on the next day of reseeding. Scale bar = 610 μm. The reseeded WT maintained its initial morphological characteristics. (C) After 2 days of reseeding, the WT organoids were passaged to demonstrate their sustainable culturability. Representative pictures were taken on Day 1 and Day 3, scale bar = 610 μm.
**Figure S3.** Rapamycin‐treated tumor organoid did not change tumor subcell types. The subcell type of organoids was further characterized by fluorescent staining. (A) Stem cells were labeled via the EdU assay (see Section 2). In the wild‐type (WT) organoid, stem cells are confined to the crypt‐like domain, whereas in both control (Ctrl)–treated and Rapa‐treated tumor groups, they were scattered throughout the organoid. (B) Paneth cells were labeled using lysozyme staining. There were Paneth cells apparent in WT, whereas no Paneth cells were found in both tumor‐Ctrl and Rapa treatment groups. Scale bar = 200 μm.
**Table S1.** All measured estimated parameters for conversion rate (*k*), area under the curve (AUC/substrate_*t*=0_), maximum signal intensity (max%), and time to reach the maximum signal intensity (*t*_max_) of Figure 2 in the main text and Figures S1 and S2. The sample pH value was measured inside the tube following the acquisition. The organoids coverage area was determined before the experiment. Polarization estimates of each sample were calculated from measurement in parallel polarization on a different system without administration to organoids (detailed in Table S2).
**Table S2.** Measured polarization ($P_{\mathrm{SS}}$) and $T_{1,\mathrm{SS}}$ on a 1T SpinSolve ^13^C machine with transfer times to the SpinSolve ($t_{\mathrm{SS}}$) and the Bruker 9.4T machine ($t_{\text{Bruker}}$) after injection of the hyperpolarized solution to the cells of Figure 2 in the main text and Figures S1 and S2. Using the $P_{\text{Bruker}}=P_{\mathrm{SS}}*\exp\left(\frac{t_{\mathrm{SS}}-t_{\text{Bruker}}}{T_{1,\mathrm{SS}}}\right)$ equation, the polarization at the time of injection to the organoids was estimated.
**Table S3.** All measured estimated parameters for conversion rate (*k*), area under the curve (AUC/substrate_*t*=0_), maximum signal intensity (max%), and time to reach the maximum signal intensity (*t*_max_) of Figure 3 in the main text. The sample pH value was measured inside the tube following the acquisition. The organoids coverage area was determined before the experiment. Polarization estimates of each sample were calculated from measurement in parallel polarization on a different system without administration to organoids (detailed in Table S2).
**Table S4.** Measured polarization ($P_{\mathrm{SS}}$) and $T_{1,\mathrm{SS}}$ on a 1T SpinSolve ^13^C machine with transfer times to the SpinSolve ($t_{\mathrm{SS}}$) and the Bruker 9.4T machine ($t_{\text{Bruker}}$) after injection of the hyperpolarized solution to the cells of Figure 3 in the main text. Using the $P_{\text{Bruker}}=P_{\mathrm{SS}}*\exp\left(\frac{t_{\mathrm{SS}}-t_{\text{Bruker}}}{T_{1,\mathrm{SS}}}\right)$ equation, the polarization at the time of injection to the organoids was estimated.

## Data Availability

The necessary raw and analyzed data sets will be available on Zenodo: https://doi.org/10.5281/zenodo.14565816.
