# Supplementary material for: Nuclear spin hyperpolarization of pyruvate enables longitudinal monitoring of treatment response in intestinal tumor organoids
Source: Magn Reson Med. 2025 Jul 30;94(6):2567–77. doi: 10.1002/mrm.70008 (PMC12501731; doi:10.1002/mrm.70008)
Supplement: Supplementary file 1 — Figure S1. Strong metabolic effect of rapamycin treatment revealed by hyperpolarization‐enhanced NMR. Four data sets were evaluated in both cases (four control, four treated with rapamycin). (A) The k‐rate for lactate as anaerobe metabolism marker is significantly lower in the treated tumor organoids (6.6 times, effect size d = 2.7). No alanine signal was observed in the treated tumor organoids, suggesting that alanine experiences also about the same difference in signal or more between control and treated when compared with lactate, as the alanine in the treated group is hidden within the noise floor (signal‐to‐noise ratio [SNR] of alanine of about 5 in the control group). Bicarbonate was only found in one instance for the treated organoids. This suggests a higher average metabolism for the control group in all cases. (B) The areas under kinetic curve (AUC) (Figure 2) closely match the results (treated 5.3 times lower, d = 3.9) from (A), as do the maximum signal intensity (C) treated 6.2‐times lower, d = 3.5). The maximum bicarbonate signal was found to be just above. Figure S2. Comparison of pyruvate to lactate conversion rate, k, in wild‐type (WT), tumor control, and tumor rapamycin‐treated organoids and their morphology. (A) Comparison of the metabolism of tumor organoids to WT organoids. The WT organoids were enriched to increase the density of cells in the Matrigel. Without enriching, we could not readily observe their metabolism using hyperpolarization. The densities inside the Matrigel were 18.4% (WT), 34.8% (control), and 21.7% (stimulated). When comparing the conversion rates of Rapa and WT groups, the k of the WT organoids was higher, despite its lower density. This indicates that the rapamycin treatment was efficiently suppressing metabolism below the level of the WT organoids, indicating the success of the treatment. Polarization across all experiments was (28.6 ± 6.0)%, and pH in the NMR tube was 7.4 ± 0.4. (B) The WT organoids were reseeded after test [file MRM-94-2567-s001.docx]

Supporting materials for

Nuclear spin hyperpolarization of pyruvate enables longitudinal monitoring of treatment response in intestinal tumor organoids

Josh P. Peters^1^*, Hang Xiang^2^*, Charbel Assaf^1^, Farhad Haj Mohamad^1^, Philip Rosenstiel^2^, Stefan Schreiber^3^, Jan-Bernd Hövener^1^, Konrad Aden^2,3†^ and Andrey N. Pravdivtsev^1†^

^1^ Section Biomedical Imaging, Molecular Imaging North Competence Center (MOIN CC), Department of Radiology and Neuroradiology, University Medical Center Kiel, Kiel University, Am Botanischen Garten 14, 24118, Kiel, Germany

^2^ Institute of Clinical Molecular Biology, Kiel University, Rosalind-Franklin-Straße 12, 24105 Kiel, Kiel, Germany

^3^ Department of Internal Medicine I, University Medical Center Kiel, Kiel, Germany

* Equal contribution

*^†^ Corresponding authors:* Dr. Andrey N. Pravdivtsev: [andrey.pravdivtsev@rad.uni-kiel.de](mailto:andrey.pravdivtsev@rad.uni-kiel.de)

Contents

[Metabolic kinetic parameters of control and rapamycin-treated tumor organoids 2](#_Toc183172204)

[Lactate production rate in WT, tumor-control, and tumor-rapamycin-treated organoids 3](#_Toc183172205)

[Phenotype behavior of organoids 4](#_Toc183172206)

[Estimated parameters of hyperpolarization and metabolic conversion 5](#_Toc183172207)

# Metabolic kinetic parameters of control and rapamycin-treated tumor organoids


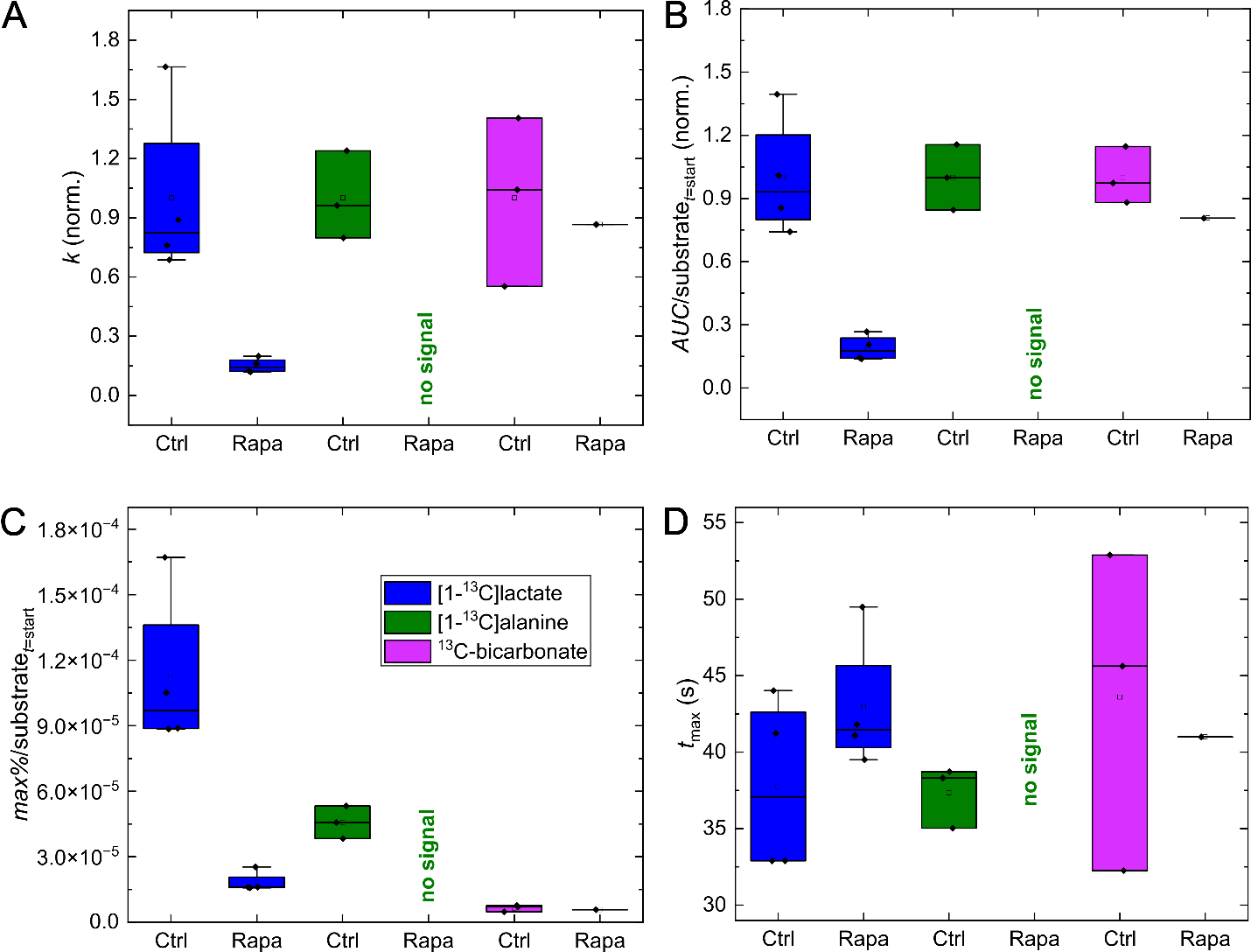


**Figure S1: Strong metabolic effect of rapamycin treatment revealed by hyperpolarization enhanced NMR.** Four datasets were evaluated in both cases (4 control, 4 treated with rapamycin). (A) The k-rate for lactate as anaerobe metabolism marker is significantly lower in the treated tumor organoids (6.6-times, effect size d=2.7). No alanine signal was observed in the treated tumor organoids suggesting that alanine experiences also about the same difference in signal or more between control and treated when compared to lactate, since the alanine in the treated group is hidden within the noise floor (SNR of alanine of about 5 in control group). Bicarbonate was only found in one instance for the treated organoids. This suggests a higher average metabolism for the control group in all cases. (B) The areas under kinetic curve (AUC, **Figure 2**) closely matches the results (treated 5.3-times lower, d=3.9) from A, as does maximum signal intensity (C, treated 6.2-times lower, d=3.5). The maximum bicarbonate signal was found to be just above the noise floor, making analysis less accurate. (D) A difference in t_max_ for lactate was observed between the treated and untreated organoids (5.2 seconds, d=1.0), though the difference is not significant. The big deviation between bicarbonate data points (SNR of only about 1.7) is not sufficient to generate quantitative results. The differences in A-C are significant to p<0.005 with (p = 0.0161) and p<0.05 without (p = 0.0047) equal variance assumed. The polarization of all experiments was (27.0±5.7)% after (29.4±12.2) s following dissolution.

# Lactate production rate in WT, tumor-control, and tumor-rapamycin-treated organoids


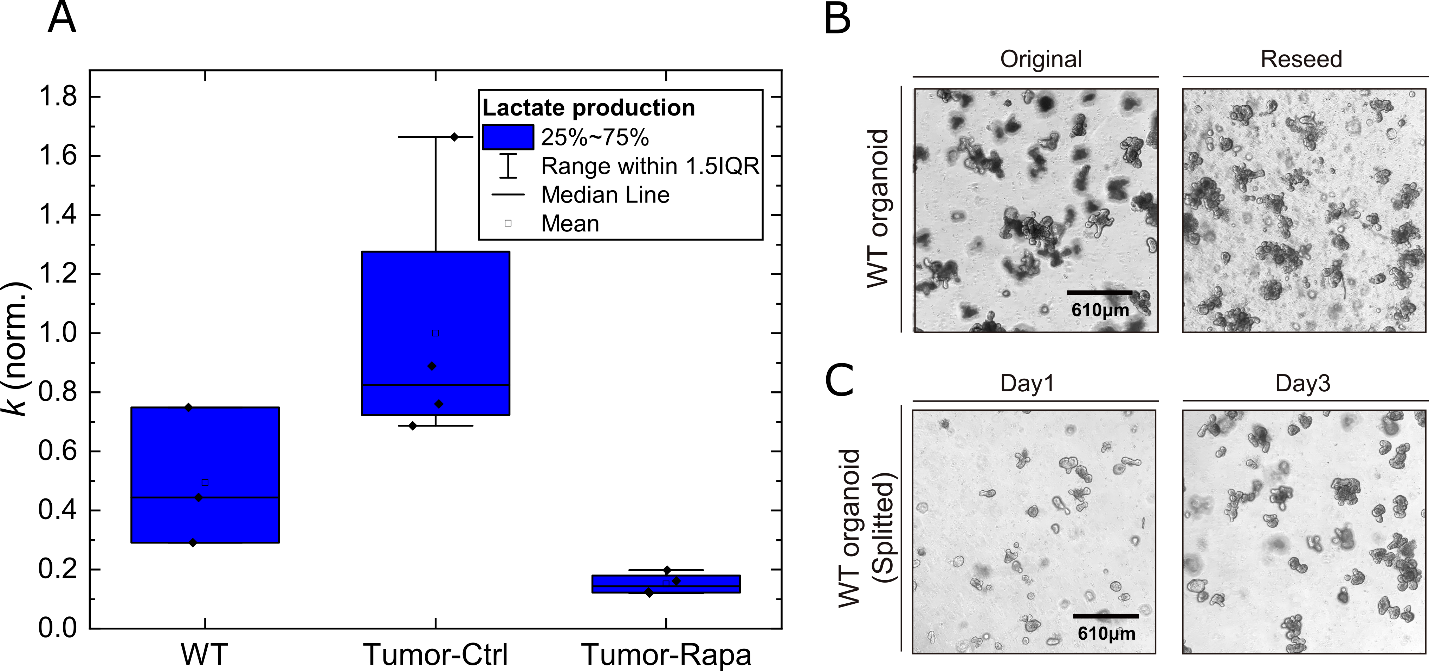


**Figure S2. Comparing pyruvate to lactate conversion rate, k, in wild-type, tumor control, and tumor rapamycin-treated organoids and their morphology.** (A) *We compared the metabolism of tumor organoids to WT organoids. The WT organoids were enriched to increase the density of cells in the Matrigel. Without enriching, we were not able to readily observe their metabolism using hyperpolarization. The densities inside the Matrigel were 18.4% (WT), 34.8% (control), and 21.7% (stimulated). When comparing the conversion rates of Rapa and WT groups, the k of the WT organoids was higher despite its lower density. This indicates that the rapamycin treatment was efficiently suppressing metabolism below the level of the WT organoids, indicating the success of the treatment.* Polarization across all experiments was (28.6±6.0)% and pH in the NMR tube was 7.4±0.4. (B*)The WT organoids were reseeded after testing and representative pictures were taken on the next day of reseeding. Scale bar = 610μm. The reseeded WT maintained its initial morphological characteristics. (C) After 2 days of reseeding, the WT organoids were passaged to demonstrate its sustainable culturability. Representative pictures were taken on Day 1 and Day 3, scale bar = 610 μm.*

# Phenotype behavior of organoids


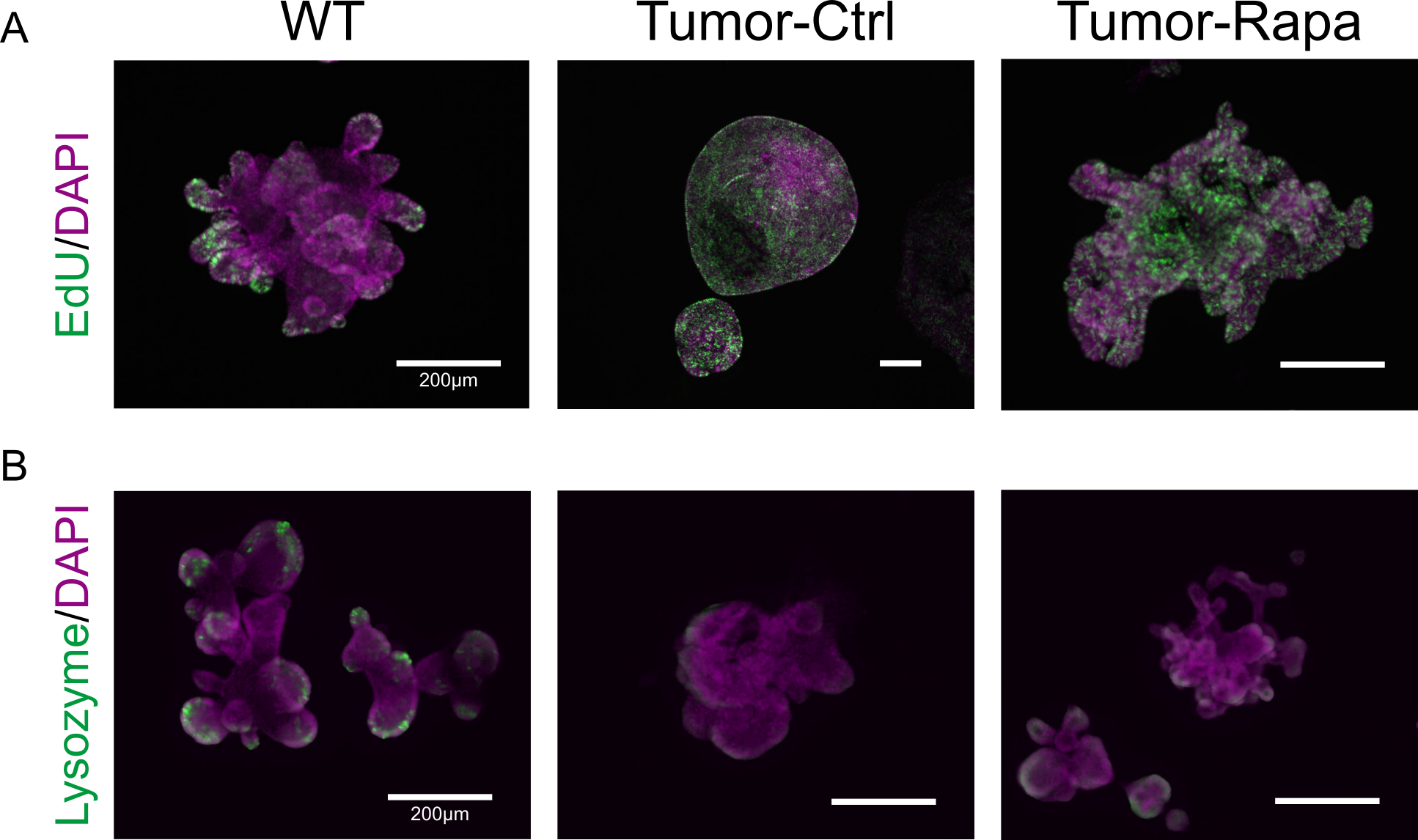


**Figure S3**. **Rapamycin treated tumor organoid did not change tumor Sub-cell types.** The sub-cell type of organoids was further characterized by fluorescent staining. (A) Stem cells were labeled via the EdU assay (see methods). In WT organoid, stem cells are confined to the crypt-like domain, whereas in both Ctrl and Rapa treated tumor groups, they are scattered throughout the organoid. (B) Paneth cells were labeled using lysozyme staining. There were Paneth cells apparent in WT, whereas no Paneth cells were found in both tumor-Ctrl and Rapa treatment groups. Scale bar = 200 μm.

# Estimated parameters of hyperpolarization and metabolic conversion

The tables below refer to the data presented in **Figure 2, Figure S1, and Figure S2**.

**Table S1.** All measured estimated parameters for conversion rate (k), area-under-the-curve (AUC/substrate_t=0_), maximum signal intensity (max%), and time to reach the maximum signal intensity (t_max_) of **Figure 2** in the main text and **Figures S1** and **S2** in SI. The sample pH value was measured inside the tube following the acquisition. The organoids coverage area was determined before the experiment. Polarization estimates of each sample were calculated from measured in parallel polarization at different system without administration to organoids (detailed in Table S2).

| Sample | Coverage area (%) | Sample pH | Polarization estimates (%) | k *10^6^ (1/s) | AUC*10^3^/  substrate_t=0_ | max%*10^2^ (%) | t_max_ (s) |
| --- | --- | --- | --- | --- | --- | --- | --- |
| Control1 | 35.1 | 7.49 | 24.9 | 14.3 | 20.4 | 1.67 | 32.9 |
| Control2 | 33.1 | 6.87 | 37.5 | 5.91 | 12.5 | 0.89 | 41.2 |
| Control3 | not performed | 7.70 | 20.1 | 6.55 | 14.7 | 1.05 | 44.0 |
| Control4 | 24.5 | 7.70 | 27.9 | 7.65 | 10.8 | 0.89 | 32.9 |
| Rapa1 | 30.6 | 7.85 | 25.3 | 1.07 | 2.11 | 0.16 | 41.1 |
| Rapa2 | 35.2 | 6.89 | 20.5 | 1.70 | 3.02 | 0.16 | 39.5 |
| Rapa3 | 21.4 | 7.24 | 25.3 | 1.39 | 3.91 | 0.25 | 49.5 |
| Rapa4 | not performed | 7.61 | 34.0 | 1.03 | 2.02 | 0.16 | 41.8 |
| WT1 | 18.0 | 7.51 | 33.0 | 6.45 | 7.83 | 0.69 | 30.4 |
| WT2 | 20.1 | 6.87 | 38.0 | 3.82 | 4.28 | 0.38 | 28.6 |
| WT3 | 17.1 | 7.66 | 27.6 | 2.50 | 3.44 | 0.28 | 32.1 |

**Table S2.** Measured polarization ($P_{\mathrm{SS}}$) and $T_{1,SS}$ on 1 T SpinSolve ^13^C machine with transfer times to the SpinSolve ($t_{SS}$) and the Bruker 9.4 T machine ($t_{\mathrm{Bruker}}$) after injection of the hyperpolarized solution to the cells) of Figure 2 in the main text and Figures S1 and S2 in SI. Using the $P_{\mathrm{Bruker}}=P_{\mathrm{SS}}*exp \left( \frac{t_{\mathrm{SS}}-t_{\mathrm{Bruker}}}{T_{1,SS}} \right)$ equation, the polarization at the time of injection to the organoids was estimated.

| Sample | Polarization SpinSolve (%) | T_1_ SpinSolve (s) | Transfer SpinSolve (s) | Transfer Bruker (s) | Polarization estimates Bruker (%) |
| --- | --- | --- | --- | --- | --- |
| Control1 | 27.71 | 54.6 | 16.5 | 22.4 | 24.87 |
| Control2 | 40.37 | 78.6 | 15.1 | 21.0 | 37.45 |
| Control3 | 35.40 | 75.9 | 16.0 | 58.8 | 20.14 |
| Control4 | 32.05 | 74.4 | 15.2 | 25.4 | 27.94 |
| Rapa1 | 31.04 | 41.0 | 16.2 | 24.5 | 25.35 |
| Rapa2 | 24.33 | 49.2 | 15 | 23.5 | 20.47 |
| Rapa3 | 34.19 | 78.4 | 14.6 | 38.2 | 25.30 |
| Rapa4 | 36.38 | 75.7 | 16.5 | 21.6 | 34.01 |
| WT1 | 34.8 | 74.8 | 16 | 20.1 | 32.95 |
| WT2 | no acquisition | no acquisition |  | 21.6 | 38.04 |
| WT3 | 29.5 | 76.8 | 16 | 21.1 | 27.64 |

The tables below refer to the data presented in **Figure 3**.

**Table S3.** All measured estimated parameters for conversion rate (k), area-under-the-curve (AUC/substrate_t=0_), maximum signal intensity (max%), and time to reach the maximum signal intensity (t_max_) of Figure 3 in the main text. The sample pH value was measured inside the tube following the acquisition. The organoids coverage area was determined before the experiment. Polarization estimates of each sample were calculated from measured in parallel polarization at different system without administration to organoids (detailed in Table S2).

| Sample | Coverage area (%) | Sample pH | Polarization estimates (%) | k *10^6^ (1/s) | AUC*10^3^/  substrate_t=0_ | max%*10^2^ (%) | t_max_ (s) |
| --- | --- | --- | --- | --- | --- | --- | --- |
| Control1 | 33.7 | 7.42 | 38.5 | 10.9 | 14.0 | 1.16 | 30.4 |
| Control1_reseed | 36.4 | 7.6 | 37.5 | 6.5 | 8.8 | 0.72 | 31.7 |
| Rapa1 | 19.8 | 7.38 | 31.1 | 4.3 | 3.3 | 0.34 | 22.4 |
| Rapa1_reseed | 37.7 | 7.74 | 36.6 | 3.8 | 5.8 | 0.45 | 32.9 |
| Control2 | 38.5 | 7.74 | 33.0 | 10.5 | 16.4 | 1.29 | 34.4 |
| Control2_reseed | 30.6 | 7.39 | 35.7 | 17.7 | 16.2 | 1.46 | 24.4 |
| Rapa2 | 19.9 | none | 33.9 | 3.5 | 5.3 | 0.43 | 34.7 |
| Rapa2_reseed | 9.4 | 7.99 | 38.3 | 1.24 | 2.1 | 0.15 | 34.3 |

**Table S4.** Measured polarization ($P_{\mathrm{SS}}$) and $T_{1,SS}$ on 1 T SpinSolve ^13^C machine with transfer times to the SpinSolve ($t_{SS}$) and the Bruker 9.4 T machine ($t_{\mathrm{Bruker}}$) after injection of the hyperpolarized solution to the cells of Figure 3 in the main text. Using the $P_{\mathrm{Bruker}}=P_{\mathrm{SS}}*exp \left( \frac{t_{\mathrm{SS}}-t_{\mathrm{Bruker}}}{T_{1,SS}} \right)$ equation, the polarization at the time of injection to the organoids was estimated.

| Sample | Polarization SpinSolve (%) | T_1_ SpinSolve (s) | Transfer SpinSolve (s) | Transfer Bruker (s) | Polarization estimates Bruker (%) |
| --- | --- | --- | --- | --- | --- |
| Control1 | 39.9 | 75.7 | 17 | 19.7 | 38.48 |
| Control1_reseed | 37.6 | 76.7 | 21 | 21.2 | 37.49 |
| Rapa1 | 33.6 | 75.9 | 17 | 22.9 | 31.11 |
| Rapa1_reseed | 38.2 | 73.3 | 21 | 20.1 | 36.61 |
| Control2 | 35.0 | 76.4 | 17 | 21.3 | 33.04 |
| Control2_reseed | 37.7 | 76.8 | 15.5 | 19.6 | 35.68 |
| Rapa2 | 34.0 | 75.4 | 24 | 24.3 | 33.91 |
| Rapa2_reseed | 40.1 | 77.6 | 16.5 | 20.0 | 38.32 |
